# A High-Performance UVA Photodetector Based on Polycrystalline Perovskite MAPbCl_3_/TiO_2_ Nanorods Heterojunctions

**DOI:** 10.3390/s23156726

**Published:** 2023-07-27

**Authors:** Yupeng Zhang, Yannan Zhai, Hui Zhang, Zhaoxin Wang, Yongfeng Zhang, Ruiliang Xu, Shengping Ruan, Jingran Zhou

**Affiliations:** 1 College of Electronic Science & Engineering, Jilin University, 2699 Qianjin Street, Changchun 130012, China; jilinzhangyupeng@sina.com (Y.Z.); 13194369969@163.com (Y.Z.); 2 Aviation University of Air Force, 7855 Renmin Street, Changchun 130012, China; zhaiyannan@sina.com (Y.Z.); zhanghuirun@163.com (H.Z.); 17704316616@163.com (Z.W.); 3 State Key Laboratory of High Power Semiconductor Lasers, School of Science, Changchun University of Science and Technology, 7089 Wei-Xing Road, Changchun 130022, China; xurl@cust.edu.cn; 4 Changchun University of Science and Technology Chongqing Research Institute, 618 Liangjiang Road, Chongqing 130022, China

**Keywords:** polycrystalline perovskite, MAPbCl_3_, TiO_2_ nanorods, heterojunction, dark current, UV photodetector, high performance

## Abstract

The application of TiO_2_ nanorods in the field of ultraviolet (UV) photodetectors is hindered by a high dark current, which is attributed to crystal surface defects and intrinsic excitation by carrier thermal diffusion. Here, a photodetector based on polycrystalline perovskite MAPbCl_3_/TiO_2_ nanorods heterojunctions has been fabricated to overcome the shortcoming. The structure was composed of horizontal MAPbCl_3_ polycrystalline and vertically aligned TiO_2_ nanorods array. Many localized depletion regions at the MAPbCl_3_/TiO_2_ interface can reduce the dark current. The TiO_2_/MAPbCl_3_ detector shows high performance including a high ratio of light-dark current of about six orders of magnitude, which is much larger than that of the TiO_2_ detector. This study indicates the potential in the TiO_2_/MAPbCl_3_ heterojunction to fabricate high-performance UV detectors.

## 1. Introduction

Ultraviolet (UV) photodetectors are widely used in ultraviolet communication, flame detection and biological cell canceration detection [1,2,3,4]. Recently, various kinds of structures for UV photodetectors have been fabricated, such as Metal-Semiconductor-Metal (MSM) [5,6,7], PN junction [8,9,10], Schottky [11,12] and heterojunction [13,14]. Among the structures above, 1D nanowires and nanorods (TiO_2_ [15], ZnO [16], CdS [17], Ga_2_O_3_ [18] and so on) have drawn considerable attention due to their significant advantages for UV photodetector performance, including the stable spatial distribution of straightforward nanochannels for electron transport and light scattering. However, the detectors often suffer from a large dark current, which increases static power consumption and decreases the ratio of photo-dark current [19,20].

Researchers have found that an advantage of the TiO_2_ nanorods array in the UV photodetector is a reduction of the recombination probability of photogenerated electron–hole pairs because of the surface trap states associated with adsorbed O_2_ molecules on the surface of TiO_2_ nanorods [21]. Hakan Karaagac et al. fabricated a Schottky UV photodetector based on well-aligned TiO_2_ nanorod arrays, which exhibits high photosensitivity and excellent spectral selectivity, but the dark current (1.2 × 10^−7^ A at reverse 1 V) was relatively high [21].

CH_3_NH_3_PbCl_3_ (MAPbCl_3_) absorbs UV light below 400 nm due to its 2.88–3.11 eV bandgap [22,23,24] and has been applied in UV detection due to its high optical absorption capacity, high carrier mobility, long carrier diffusion length and stability. UV detectors based on MAPbCl_3_ single crystal show high properties, but the difficult fabrication technique places a restriction on their development in photonic crystal structures [25,26,27]. Therefore, polycrystalline MAPbCl_3_ film is preferred to compose the heterojunction. Jialin Yang et al. have successfully studied how a polycrystalline CH_3_NH_3_PbCl_3_/ZnO heterojunction improved UV photodetector performance compared to single ZnO, but the dark current remained high [28]. Liu shuo et al. have fabricated a Ga_2_O_3_/polycrystalline MAPbCl_3_ UVA photodetector with rapid response and recovery property, which nonetheless had a dark current of 6.8 μA at −1.5 V bias [29].

In this work, a UV photodetector based on polycrystalline perovskite MAPbCl_3_/TiO_2_ nanorods array heterojunction has been successfully prepared. The UV photodetector with many localized depletion regions at the MAPbCl_3_/TiO_2_ interface shows better performance than that of pure TiO_2_, with improved dark current, light-dark current ratio and a shorter response time. The results indicate that MAPbCl_3_/TiO_2_ heterojunction is a promising way to improve UV photodetector performance.

## 2. Materials and Methods

### 2.1. Preparation of TiO_2_ One-Dimensional Nanorods Array Film on FTO

TiO_2_ nanorods array film was prepared on the surface of the FTO (fluorine-doped tinoxide) substrate by a low-temperature hydrothermal method. First, FTO substrate (15 Ω per square) was cleaned in acetone, ethanol and deionized water and dried in a nitrogen stream. Subsequently, 10 mL of toluene, 1 mL of tetrabutyltitanate [Ti(OC_4_H_9_)_4_], 0.2 mL of titanium tetrachloride and 1 mL hydrochloric acid (37%) were added in a sealed Teflon-lined stainless steel autoclave (23 mL). Then, the substrate was placed in the autoclave, heated at 150 °C for 5 h and air-cooled to room temperature (25 °C). After washing with deionized water, a uniform nanorods array was obtained. The TiO_2_ growing system in this experiment is a mixed solution composed of Ti(OC_4_H_9_)_4_, Ti(OH)_4_, HCl and H_2_O. Ti(OC_4_H_9_)_4_ is a lipid with a boiling point of 310 °C. Due to the effect of HCl, Ti(OC_4_H_9_)_4_ does not hydrolyze at room temperature, but it will hydrolyze with water at high temperature and pressure. The polar H_2_O was adsorbed on the transparent, which is a conductive glass substrate of FTO with the same polarity. Ti(OC_4_H_9_)_4_ then moved to the surface of FTO and hydrolyzed with H_2_O to generate Ti(OH)_4_. Then, Ti(OH)_4_ combined with each other and underwent a polycondensation reaction to generate TiO_2_ and a small amount of H_2_O. The TiO_2_ nanorods grew firmly on the surface of FTO and the small amount of H_2_O generated by the condensation polymerization reaction continued to adsorb on the FTO surface or on the grown hydrophilic TiO_2_ layer, like other H_2_O. The reaction continued until the TiO_2_ nanorods array film generated on FTO [30].

### 2.2. Preparation of TiO_2_ Nanorods/MAPbCl_3_ Heterojunction on FTO

Polycrystalline MAPbCl_3_ film was prepared by a one-step spin-coating method with antisolvent-assisting. First, 1 mL dimethyl sulfoxide (DMSO) and 1 mL N, N-Dimethylformamide (DMF) were mixed, and then 0.135 g MACl and 0.566 g PbCl_2_ were weighed and added to the above solution and stirred for 30 min until the solution became completely transparent to obtain MAPbCl_3_ precursor solution (1 mol/L) [23]. Then, 75 μL solution was coated on TiO_2_ film by rotation for 30 s at 3000 rpm. Next, a drop of toluene was put on the TiO_2_ film and spun for 20 s. Finally, it was dried at 80 °C for 5 h. Finally, two Ag paste pads were deposited directly on the film and FTO, respectively, to make a Schottky photodiode UV detector. The active area of the electrode is about 0.25 mm^2^.

### 2.3. Material Characterization and Device Measurement

The morphology of the prepared films was characterized by a scanning electron microscope (SEM JEOS JSM-6700F). X-ray diffraction (XRD) patterns were performed using a Shimadzu XRD-6000 diffractometer (Shimadzu, Kyoto, Japan). A Shimadzu UV-3600 Pharma Spec UV spectrophotometer was used to obtain the UV-Vis absorption spectra. The photoelectric performances were analyzed by a program-controlled semiconductor characterization system (Keithley 2450 Source Meter, Solon, OH, USA). The light source was provided by a 30 W deuterium lamp, and a monochromatic lamp was used to provide monochromatic light.

## 3. Results and Discussion

**Figure 1:**
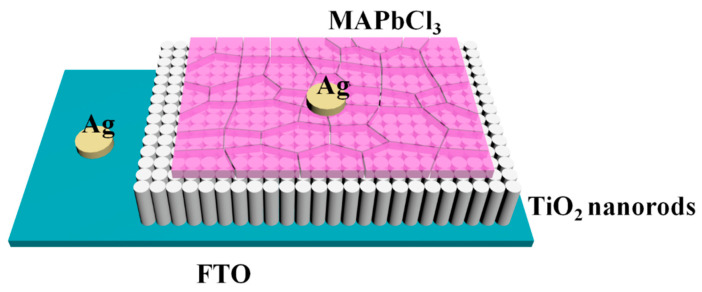
Structure of the TiO_2_ nanorods array/MAPbCl_3_ heterojunction photodetector.

The UVA photodetector with the structure of FTO/TiO_2_ nanorods array/polycrystalline perovskite MAPbCl_3_ is shown in Figure 1. TiO_2_ nanorods/perovskite MAPbCl_3_ heterojunctions worked as the active layer. FTO and Ag served as electrodes (A mask plate with a hollow circular pattern (about 0.25 mm^2^) was used to mask the material. Silver paste electrodes were coated on the FTO and the material, respectively, and the leads were led out for testing. Then, the whole device was dried at 75 °C for 15 min to stabilize the electrodes. Then, two red and black wires were led out from the Keithley 2450, with the red wire (positive) connected to the Ag above the FTO and the black wire (negative) connected to the Ag above the semiconductor).

**Figure 2:**
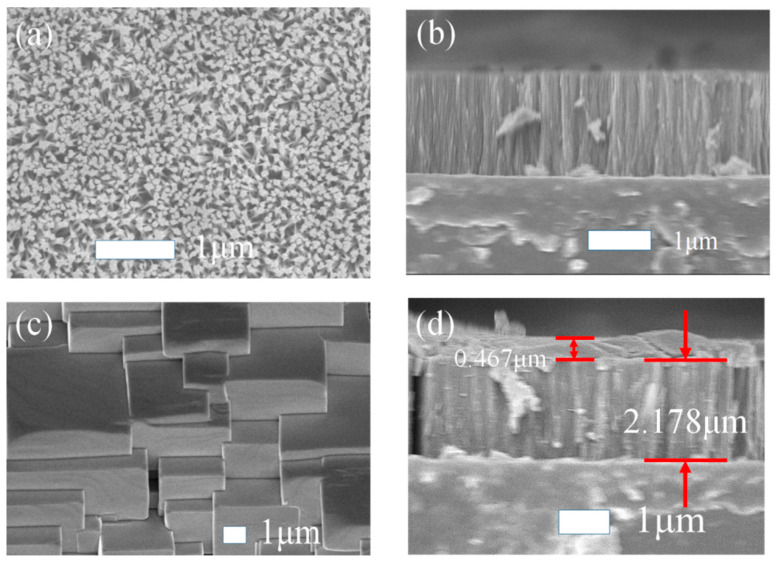
(**a**) Surface SEM image of TiO_2_ one-dimensional nanorods array film on FTO. (**b**) Cross-sectional SEM image of TiO_2_ one-dimensional nanorods array film on FTO. (**c**) Surface SEM image of TiO_2_/MAPbCl_3_ heterojunction. (**d**) Cross-sectional SEM image of TiO_2_/MAPbCl_3_ heterojunction.

The morphology of TiO_2_ one-dimensional nanorods array film on FTO and TiO_2_ nanorods/MAPbCl_3_ heterojunctions on FTO is shown in Figure 2. Figure 2a,b show the top-view and the sectional-view SEM images of the TiO_2_ one-dimensional nanorods array, respectively. It can be observed that the TiO_2_ nanorods array is uniform, compact and perpendicular to the substrate, which is conducive to the preparation of electronic devices. Figure 2c,d show the top-view and the sectional-view SEM images of the TiO_2_ nanorods/MAPbCl_3_ heterojunctions, respectively. It can be found that the polycrystalline MAPbCl_3_ film prepared by the one-step method covers the surface of TiO_2_ nanorods well. The thickness of MAPbCl_3_ film is about 0.467 μm, and the thickness of TiO_2_ nanorods film is about 2.178 μm. There are lots of grain boundaries in polycrystalline MAPbCl_3_, which could scatter carriers and thus lead to low mobility. The MAPbCl_3_ layer adheres to the TiO_2_ nanorods array layer, which contributes to the high performance of the detector.

**Figure 3:**
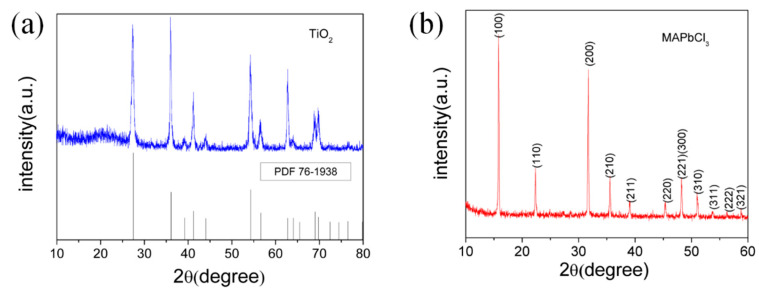
XRD patterns of (**a**) TiO_2_ nanorods and (**b**) polycrystalline perovskite MAPbCl_3_ film.

In Figure 3, which displays the XRD patterns of the TiO_2_ nanorods array and polycrystalline perovskite MAPbCl_3_, it can be observed that the crystal diffraction peaks are very high, indicating that the obtained materials have good crystallization and clear crystal particles. Figure 3a shows that the diffraction peaks of the obtained TiO_2_ correspond exactly to the peaks of the standard rutile type TiO_2_ (JCPDS Card No.76-1938). The characteristic diffraction peaks of MAPbCl_3_ generated by crystal planes (100), (110), (200), (210) and (211) in Figure 3b are included, indicating that the prepared MAPbCl_3_ perovskite shows consistency with the previously reported data of MAPbCl_3_ perovskite obtained through the conventional crystallization technique [31].

**Figure 4:**
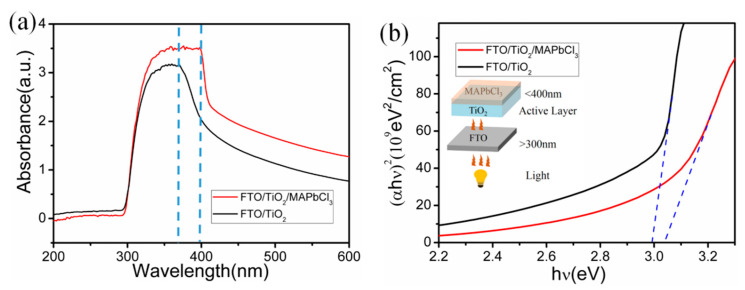
(**a**) UV-visible absorption spectra of FTO/TiO_2_ and FTO/TiO_2_/MAPbCl_3_. (**b**) Tauc plots of FTO/TiO_2_ and FTO/TiO_2_/MAPbCl_3_. The inset is a schematic diagram of light transmission.

The optical characteristics of each layer in the photodetector were studied by absorption spectra and Tauc plots, as shown in Figure 4. Figure 4a shows the UV visible absorption spectra of FTO/TiO_2_ and FTO/TiO_2_/MAPbCl_3_ from 300 nm to 600 nm. When the photodetector operates, the illumination light enters from the FTO side. Radiation with a wavelength below 300 nm is completely absorbed, and only radiation with a wavelength above 300 nm can pass through the FTO substrate. Moreover, the active layer composed of TiO_2_ or TiO_2_/MAPbCl_3_ absorbs radiation with a wavelength below 400 nm. TiO_2_ has excellent absorption, from 330 to370 nm, and the absorption decreases over 370 nm, while TiO_2_/MAPbCl_3_ has excellent absorption, from 330 to400 nm, and the absorption decreases over 400 nm. One of the advantages of TiO_2_/MAPbCl_3_ is that it increases the detector’s response range closer to 400 nm. Therefore, the radiation whose wavelengths distribute between 300 nm and 400 nm can be collected by TiO_2_/MAPbCl_3_, as shown in the inset of Figure 4b, which corresponds to the UVA range. As calculated in Figure 4b Tauc plots, the bandgap width of rutile TiO_2_ is 3.05 eV and the bandgap width becomes 2.98 eV when introducing MAPbCl_3_.

The absorption edge of TiO_2_/MAPbCl_3_ shows a slighter redshift than that of TiO_2_, indicating a narrower bandgap of TiO_2_/MAPbCl_3_, which will provide a possibility to adjust the detector’s response range. The practical absorption edge of TiO_2_ was lower than the theoretical value of pure rutile TiO_2_ (1240/3.0 = 413 nm). This may be attributed to the size quantization of nanorods, which has been demonstrated by Brus [32]:

(1) $$\Delta E_{g}=\frac{\pi^{2}h^{2}}{2R^{2}}\left(\frac{1}{m_{e}^{*}}+\frac{1}{m_{h}^{*}}\right)-\frac{1.82e^{2}}{\varepsilon_{R}}+polarizaitonterms$$

where *R* is the radius of semiconductor particle, $m_{e}^{*}$ and $m_{h}^{*}$ are effective masses of the electron and hole in the semiconductor, $\varepsilon_{R}$ is the permittivity of rutile TiO_2_, h is the Planck constant, $\Delta E_{g}$ is the bandgap difference between the original bulk size and nanoscale of the same semiconductor material. According to this formula, the absorption edge will move to the short wavelength as the particle size decreases [33].

**Figure 5:**
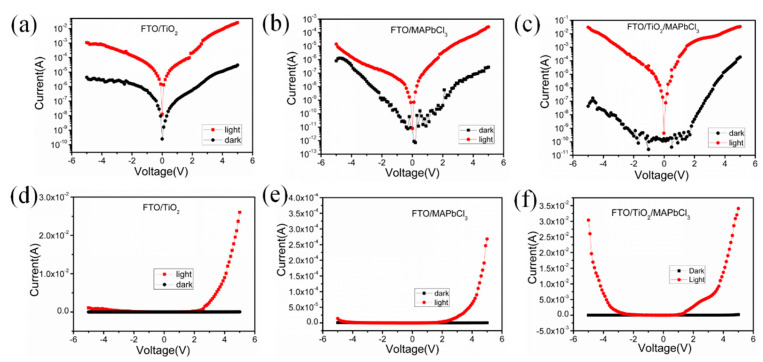
I–V characteristics of the UV detector based on (**a**) FTO/TiO_2_, (**b**) FTO/MAPbCl_3_ and (**c**) FTO/TiO_2_/MAPbCl_3_ in dark and under illumination in logarithmic coordinates; (**d**) FTO/TiO_2_, (**e**) FTO/MAPbCl_3_ and (**f**) FTO/TiO_2_/MAPbCl_3_ in dark and under illumination in linear coordinates.

The I–V characteristics of the UV detector based on FTO/TiO_2_, FTO/MAPbCl_3_ and FTO/TiO_2_/MAPbCl_3_ in dark and under illumination are shown in Figure 5. The prepared UV detectors have the characteristics of Schottky diodes, which exhibit nonlinear and unsaturated behavior, as is shown in Figure 5d–f. Under forward bias, the dark current increases rapidly and results in large noise. Therefore, we focus on the reverse characteristics of the detector. For the UV detector based on FTO/TiO_2_, at −2 V bias, the dark current is 1.557 × 10^−6^ A, and the photocurrent reaches 1.359 × 10^−4^ A under the irradiation of 200 μW/cm^2^ at a wavelength of 350 nm UV light, as shown in Figure 5a. For the UV detector based on FTO/MAPbCl_3_, at −2 V bias, the dark current is 1.043 × 10^−9^ A, and the photocurrent reaches 1.561 × 10^−7^ A under the irradiation of 580 μW/cm^2^ at a wavelength of 390 nm UV light, as shown in Figure 5b. For the UV detector based on FTO/TiO_2_/MAPbCl_3_, at −2 V bias, the dark current is 2.69 × 10^−10^ A and the photocurrent reaches 1.632 × 10^−4^ A under the irradiation of 255 μW/cm^2^ at a wavelength of 360 nm UV light, as shown in Figure 5c. The ratio of light to dark current is more than six orders of magnitude, which proves that the optical properties of the TiO_2_/MAPbCl_3_ heterojunction detector meet our requirements.

**Figure 6:**
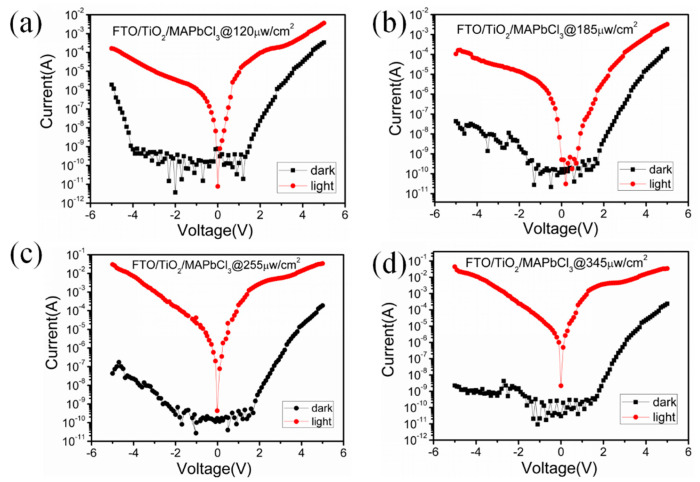
I–V characteristics of TiO_2_/MAPbCl_3_ UV detector in dark and under illumination in logarithmic coordinates with various irradiation (**a**) 120 μW/cm^2^, (**b**) 185 μW/cm^2^, (**c**) 255 μW/cm^2^ and (**d**) 345 μW/cm^2^.

Figure 6 shows I–V characteristics of FTO/TiO_2_/MAPbCl_3_ UV detector in dark and at a wavelength of 360 nm UV light with various light intensity. All data were obtained at −2 V bias. Under the irradiation of 120 μW/cm^2^, the dark current is 3.69 × 10^−12^ A and the photocurrent reaches 3.621 × 10^−6^ A. Under the irradiation of 185 μW/cm^2^, the dark current is 2.27 × 10^−9^ A and the photocurrent reaches 1.37 × 10^−5^ A. Under the irradiation of 255 μW/cm^2^, the dark current is 2.69 × 10^−10^ A and the photocurrent reaches 1.632 × 10^−4^ A. Under the irradiation of 345 μW/cm^2^, the dark current is 1.516 × 10^−9^ A and the photocurrent reaches 2.484 × 10^−4^ A. Compared with dark current of FTO/TiO_2_ UV detector, which is 10^−6^ A, the dark current of FTO/TiO_2_/MAPbCl_3_ UV detector improved a lot, which changes from 10^−9^ A to 10^−12^ A. The photo current increased as irradiation increased.

**Figure 7:**
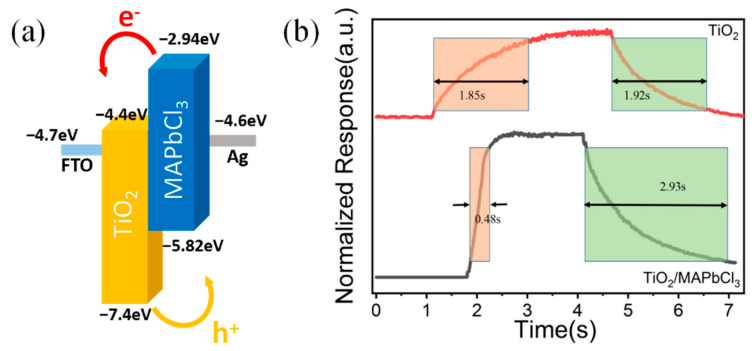
(**a**) The energy level diagrams of TiO_2_/MAPbCl_3_ heterojunction. (**b**) Rise times and decay times of pure TiO_2_ detector and TiO_2_/MAPbCl_3_ detector.

The introduction of TiO_2_/MAPbCl_3_ heterojunction plays an important role in light-to-dark current ratio. The only difference between FTO/TiO_2_ and FTO/TiO_2_/MAPbCl_3_ detectors is the TiO_2_/MAPbCl_3_ active layer in the latter. The energy level diagrams and the schematic band diagrams of TiO_2_/MAPbCl_3_ heterojunction are shown in Figure 7a [24,34]. A built-in electric field would be formed at the TiO_2_/MAPbCl_3_ interface [2,35,36]. The TiO_2_/MAPbCl_3_ detector has characteristics similar to Schottky diode. That is, at forward bias, the built-in electric field is weakened and current increases. At reversed bias, the built-in electric field is enhanced and the current reduces. Compared to the TiO_2_ detector, the dark current of TiO_2_/MAPbCl_3_ detector was reduced due to the built-in electric field of the heterojunction. Furthermore, the TiO_2_/MAPbCl_3_ contact interface between horizontal MAPbCl_3_ polycrystalline and vertically aligned TiO_2_ nanorods array can produce many localized depletion regions, which contribute to lower dark current. What’s more, grain boundaries in polycrystalline MAPbCl_3_ could scatter carriers in the dark and thus lower mobility. Therefore, the dark current of TiO_2_/MAPbCl_3_ heterojunction UV detector is much lower than that of pure TiO_2_ UV detector. Under illumination, photogenerated electron–hole pairs can be excited in two materials, which are separated by the built-in electric field at the TiO_2_/MAPbCl_3_ interface. Electrons flow along the vertical downward direction of the TiO_2_ nanorods, while holes are along the plane direction of the MAPbCl_3_ film, so it is difficult for recombination and the collection efficiency of carriers improves. Halogen interstitial defects or MA on halogen antisite defects in polycrystalline MAPbCl_3_ create deep level defects that can trap holes and can be recombination centers affected by the nonequilibrium carriers [37]. Therefore, under illumination, compared with a pure TiO_2_ UV detector, TiO_2_/MAPbCl_3_ UV detector produces more photogenerated carriers [38,39].

Figure 7b displays the response and recovery characteristics of the detectors at −2 V bias, obtained from measuring the voltage variation of a 1 MΩ load resistance in the test circuit. The rise times of the TiO_2_ detector and TiO_2_/MAPbCl_3_ detector are 1.85 s and 0.48 s, respectively, and the fall times are 1.92 s and 2.93 s, respectively. The reason why there is an improvement in response characteristic is that the photogenerated electron–hole pairs are rapidly separated by the built-in electric fields of many localized heterojunction regions, as mentioned above. As for the TiO_2_/MAPbCl_3_ photodetector, more photogenerated carriers make the recovery process slower.

**Figure 8:**
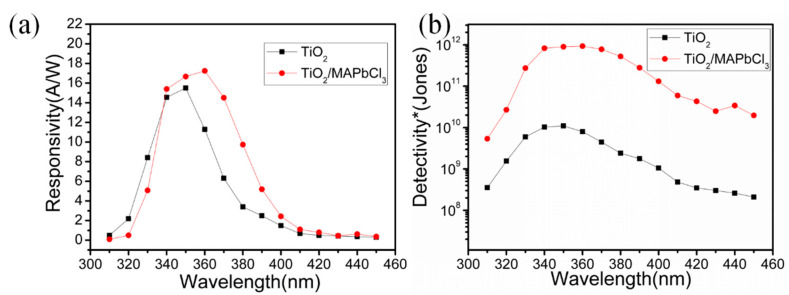
(**a**) The spectral responses of pure TiO_2_ and TiO_2_/MAPbCl_3_ heterostructure detectors. (**b**) Spectral Detectivity* of pure TiO_2_ and TiO_2_/MAPbCl_3_ heterostructure detectors.

Another important parameter for UV photodetector is spectral responsivity and Detectivity* under the irradiation of monochromatic UV light ranging from 310 nm to 450 nm. The spectral responsivity and Detectivity* of both detectors at −2 V bias are shown in Figure 8. The responsivity R was calculated by [32]:

(2) $$R=\frac{I_{p}}{A\times E}$$

in which E is the incident optical power, A is the effective photosensitive area of detector and $I_{p}$ is the photocurrent of the detector under irradiation of the corresponding incident light. The Detectivity* $D^{*}$, which demonstrates the ability to detect weak signals from a noise environment, is calculated by [40]:

(3) $$D^{*}=\frac{R}{{\left(2eI_{d}/A\right)}^{\frac{1}{2}}}$$

where e is the electronic charge constant, and $I_{d}$, which contributes to background noise, is dark current at −2 V bias. Both TiO_2_ and TiO_2_/MAPbCl_3_ detectors exhibit well spectrum selectivity for 310~450 nm and R and $D^{*}$ have increased when compared with the pure TiO_2_ device. The response peak of TiO_2_/MAPbCl_3_ detector is 17.25 A/W at 360 nm, and the corresponding $D^{*}$ is 9.2094 × 10^11^ Jones, which is higher than 15.5 A/W and the corresponding $D^{*}$ 1.097 × 10^10^ Jones of pure TiO_2_ detector at 350 nm, respectively.

The gain G represents the number of detected charge carriers per single incident photon, and is given by [41]:

(4) $$G=\frac{I_{p}/q}{\eta P_{in}/h\upsilon }$$

where η is quantum efficiency and hυ is the excitation energy. Taking TiO_2_/MAPbCl_3_ detector for example, at −2 V bias and at 360 nm UV light, the photocurrent is 1.632 × 10^−4^ A and the irradiation is 255 μW/cm^2^. The energy of each photon is 1240/360 = 3.44 eV. The incident light energy is 255 × 0.25 × 10^−2^ = 0.6375 μW. The number of photons per second falling on the unit area of the device is 0.6375 μW/(1.602 × 10^−19^ × 3.44) = 1.157 × 10^12^. Assuming that all photons are absorbed by the semiconductor surface, the photocurrent generated by these photons is supposed to be 1.157 × 10^12^ × 1.602 × 10^−19^ = 1.853 × 10^−7^ A, so the gain of TiO_2_/MAPbCl_3_ detector is 1.632 × 10^−4^/1.853 × 10^−7^ = 881. The gain of TiO_2_ detector is 963. Both of these detectors have a large gain. Under illumination, the defects of the metal/semiconductor interface will act as minority traps, thus mirroring an equal amount of opposite charges inside the semiconductor and reducing the Schottky barrier height. Therefore, more carriers pass through the barrier and obtain high photocurrent and responsivity, which leads to a large gain. The reason why the gain of TiO_2_/MAPbCl_3_ heterojunction is less than pure TiO_2_ is because the detecting wavelength (360 nm) and the corresponding irradiation power (255 μW/cm^2^) of TiO_2_/MAPbCl_3_ is larger than that of TiO_2_ (wavelength (350 nm), the corresponding irradiation power (200 μW/cm^2^)). After being calculated according to Formula (4) above, the gain of heterojunction is lower.

**Figure 9:**
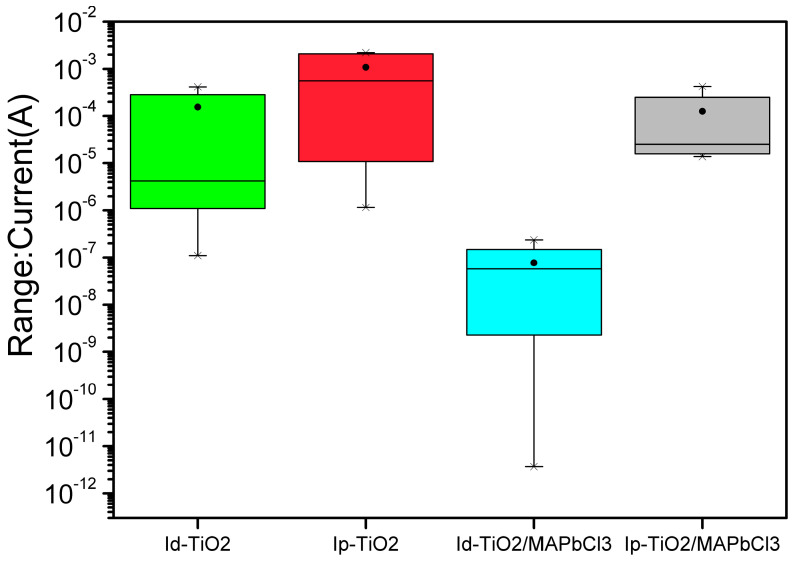
The box plots of the TiO_2_ and TiO_2_/MAPbCl_3_ photodetectors’ photo and dark currents at reverse 2 V bias.

The statistical results of the photodetectors’ performance parameters are given in Figure 9. The photo and dark currents at −2 V bias of TiO_2_ and TiO_2_/MAPbCl_3_ photodetectors are shown in box plots. We have fabricated about eight TiO_2_ detectors, and two detectors have good performance like the results above. For example, the ratio of light to dark current at −2 V bias is bigger than two orders of magnitude, and the ratio of others is smaller towards one order of magnitude. The smallest dark current at −2 V bias is close to 10^−6^ A. The reason why the performance of TiO_2_ detectors is not stable is due to fabrication process of the devices. In this work, the FTO substrate we used is 1.5 cm × 4 cm so as to be put within the 25 mL autoclave and be faced down. Then TiO_2_ nanorods array film can grow on the conductive layer of FTO during a hydrothermal process. Usually, this film covers all the area of the conductive layer, and there are lots of defects in TiO_2_ nanorods film, which leads to relatively high dark and photo currents. If the TiO_2_ film didn’t form well on FTO substrate, the performance of UV detector would be poor.

We have fabricated about fifteen TiO_2_/MAPbCl_3_ detectors and four of them have good performance. That is, the ratio of light to dark current at −2 V bias is bigger than six orders of magnitude, and the ratio of others is smaller from four to two orders of magnitude. The smallest dark current at −2 V bias is close to 10^−11^ A. Because the perovskite MAPbCl_3_ might not be stable in the atmosphere (H_2_O, O_2_), and the fabrication process of MAPbCl_3_ might also influence the quality of film, the performance of TiO_2_/MAPbCl_3_ detectors is not stable compared to our other device with different structures and fabrication procedure (such as MSM detectors with sol-gel method). We have fabricated many detectors and choose the detectors with relatively good performances.

Table 1 lists the dark current and responsive performances comparison between some reported TiO_2_ based UV detectors. It is clear that the TiO_2_/MAPbCl_3_-based UV detector shows a high responsivity and detectivity*, a fast response speed and a low dark current.

## 4. Conclusions

A high-performance UVA photodetector based on a polycrystalline perovskite MAPbCl_3_/TiO_2_ nanorods heterojunction has been fabricated successfully. MAPbCl_3_ polycrystalline perovskite film forms a good heterojunction with TiO_2_ one-dimensional nanorods by one-step spin-coating method with antisolvent-assisting. The special structure makes the TiO_2_/MAPbCl_3_ contact interface produce plenty of localized depletion regions. Responsivity and response properties were also improved. Therefore, we successfully improved the performance of pure TiO_2_ UV photodetector by introducing a TiO_2_/MAPbCl_3_ heterojunction. These results indicate that the TiO_2_/MAPbCl_3_ heterojunction detector is a potential candidate for UV detection.

## Figures and Tables

**Table 1:** Comparison chart of recent achievements in relation to TiO_2_-based UV detectors.

Materials	Fabrication Technique	Dark Current (μA)	Λ (nm)	Responsivity (A/W)	Detectivity* (Jones)	Rise Time (s)	Fall Time (s)	Ref
TiO_2_/CuI	nanorods array	4.10 × 10^−4^ A at 0 V	410	4.5 × 10^−3^	1.08 × 10^11^	0.33	0.22	[42]
TiO_2_/3-BiOCl	nanotube	7.49 × 10^−3^ A at −5 V	350	7.92	1.42 × 10^13^	17.3	1.68	[43]
TiO_2_/MoO_3_	Sol-gel method	2.856 at −1 V	352	108 × 10^−3^	2.26 × 10^10^	1.82	1.42	[44]
Ga_2_O_3_/MAPbCl_3_	amorphous	6.8 at −1.5 V	398	4.96 × 10^−3^	5.4 × 10^10^	3.21	0.067	[29]
TiO_2_/MAPbCl_3_	nanorods array	2.69 × 10^−4^ at −2 V	360	17.25	9.2094 × 10^11^	0.48	2.93	This Work

## Data Availability

The data presented in this study are available on request from the corresponding author.
